# Dealing with a hairy beast–larval morphology and chaetotaxy of the Australian endemic diving beetle genus *Spencerhydrus* (Coleoptera, Dytiscidae, Cybistrini)

**DOI:** 10.3897/zookeys.884.38391

**Published:** 2019-10-30

**Authors:** Mariano C. Michat, Yves Alarie, Chris H.S. Watts

**Affiliations:** 1 University of Buenos Aires, Faculty of Exact and Natural Sciences, Department of Biodiversity and Experimental Biology, Laboratory of Entomology, Buenos Aires, Argentina; 2 CONICET-University of Buenos Aires, Institute of Biodiversity and Experimental and Applied Biology, Buenos Aires, Argentina; 3 Department of Biology, Laurentian University, Ramsey Lake Road, Sudbury, Ontario, Canada; 4 South Australian Museum, North Terrace, Adelaide, SA 5000, Australia

**Keywords:** Water beetle, sensilla, larva, morphometry

## Abstract

In this contribution, the larval morphology of *Spencerhydrus* Sharp, 1882 was studied, an Australian endemic genus in the diving beetle tribe Cybistrini. All instars of the only two species included in the genus (*S. latecinctus* Sharp, 1882 and *S. pulchellus* Sharp, 1882) are described and illustrated with the exception of the third instar of *S. latecinctus*. Detailed morphometric and primary chaetotaxic analyses were performed to discover useful characters for generic diagnosis and species distinction. *Spencerhydrus* can be distinguished from other Cybistrini genera by the medial projection of frontoclypeus slightly indented apically, with lamellae clypeales directed forward in a characteristic V-shaped pattern, the median process of prementum strongly developed, the presence of a single ventral sclerite on prothorax, the presence of basoventral spinulae on claws, and the reduced sclerotization of the abdominal segment VII which covers only the anterior half. Larvae of the two species of *Spencerhydrus* can readily be distinguished by the shape of the median process of prementum, which is visibly broader in *S. pulchellus* than in *S. latecinctus*.

## Introduction

*Spencerhydrus* Sharp, 1882 is a small, Australian endemic genus of large diving beetles (adult length 17–18 mm) included in the tribe Cybistrini. It is made up of two species restricted to southern Australia and with clearly separated distributions, *S. latecinctus* Sharp, 1882 in the south-east, and *S. pulchellus* Sharp, 1882 in the south-west (Watts 1978). Biological information for the genus is scarce; adults are rarely collected, mainly in lentic habitats with considerable emergent vegetation (Miller and Bergsten 2016). Recent studies based mainly on adult and molecular characters placed *Spencerhydrus* in a clade of Australian cybistrines together with *Austrodytes* Watts, 1978, *Onychohydrus* Schaum & White, 1847, and *Sternhydrus* Brinck, 1945 (Miller et al. 2007; Miller and Bergsten 2014). Studies of larval characters (Michat et al. 2015, 2017) supported this placement, although *Austrodytes* was not included because its larva is unknown. Even though larval characters of *Spencerhydrus* were included in these phylogenetic analyses, larvae were not described or illustrated, and therefore morphology of members of this genus remains little known.

The only treatment of larvae of *Spencerhydrus* found in the literature is Watts (2002), in which the genus was included in a key to larvae of Australian Dytiscidae and illustrated with a photograph of the head and a drawing of the last two abdominal segments. A thorough morphometric and chaetotaxic treatment of dytiscid larvae, as that developed in the past three decades to complement the more traditional morphological study (see Alarie and Michat 2014 for a review), allows the discovery of useful characters at various taxonomic levels both for the diagnosis of taxa and for phylogenetic studies. In this context, we provide detailed descriptions and illustrations of the two species of *Spencerhydrus* with an emphasis on morphometry and chaetotaxy. With this treatment, we aim to recognize suitable characters to distinguish larvae of this genus from those of other cybistrine genera, and also to differentiate the larvae of its two known species, i.e., *S. latecinctus* and *S. pulchellus*.

## Materials and methods

Larvae were first cleared by submerging them for several days in lactic acid, then dissected in the standard way and mounted on slides with polyvinyl-lacto-glycerol as the medium. Examination (at magnifications up to 1,000×) and drawings were made using an Olympus CX31 (Olympus Corporation, Japan) compound microscope equipped with a camera lucida. Drawings were scanned and digitally inked using a Genius PenSketch tablet (KYE Corporation, Taiwan). After study, the material will be held in the collection of the South Australian Museum.

The methods and terms used herein follow those employed in Michat (2006, 2010), Alarie et al. (2011), and Michat et al. (2015). The reader is referred to those papers for a complete list and additional explanations of the terms used here. The criterion of similarity of position (Wiley 1981) was primarily used to propose homology hypotheses. It is worth mentioning, however, that larvae of Cybistrini (and of *Spencerhydrus* in particular) bear numerous additional sensilla (i.e., those evolved secondarily in the first instar) that obscure the establishment of positional homologies with the ancestral systems of other Dytiscidae. Alarie et al. (2011) showed that first instars of Cybistrini are characterized by the presence of multifid setae (i.e., setae that are split into two or more branches beyond the base), which are distributed similarly to the ancestral pattern of primary setae depicted for the subfamily Dytiscinae. The presence of a multifid seta was therefore considered as an additional argument for homology when more than one seta of *Spencerhydrus* larvae was potentially homologous with a given seta of other dytiscine genera. The ancestral chaetotaxy pattern thus established for *Spencerhydrus* is in good agreement with that observed in other genera (Alarie et al. 2011).

## Results

### Description of the larvae of *Spencerhydrus* Sharp, 1882

#### Spencerhydrus

Taxon classificationAnimaliaColeopteraDytiscidae

Sharp, 1882

E59EFF56-58EC-50B8-B105-0B2ACD410B26

##### Diagnosis.

**Figures 1–4.**
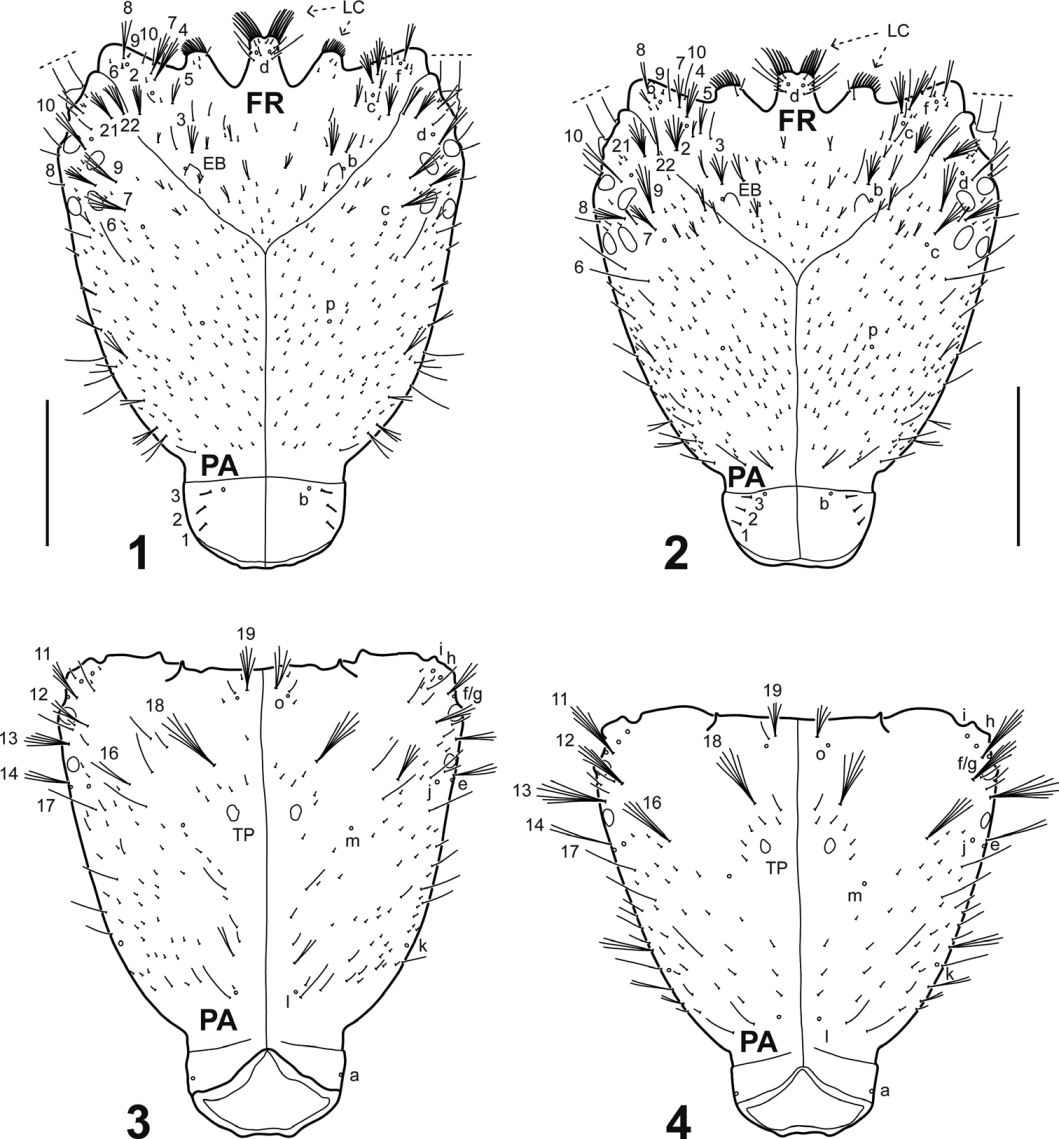
Cephalic capsule of *Spencerhydrus* species, instar I **1***S. latecinctus*, dorsal aspect **2***S. pulchellus*, dorsal aspect **3***S. latecinctus*, ventral aspect **4***S. pulchellus*, ventral aspect. Numbers and lowercase letters indicate primary setae and pores, respectively. Additional setae not labelled. Color patterns not represented. Abbreviations: EB: egg burster; FR: frontoclypeus; PA: parietal; LC: lamellae clypeales; TP: tentorial pit. Scale bar: 0.70 mm.

**Figures 5–14.**
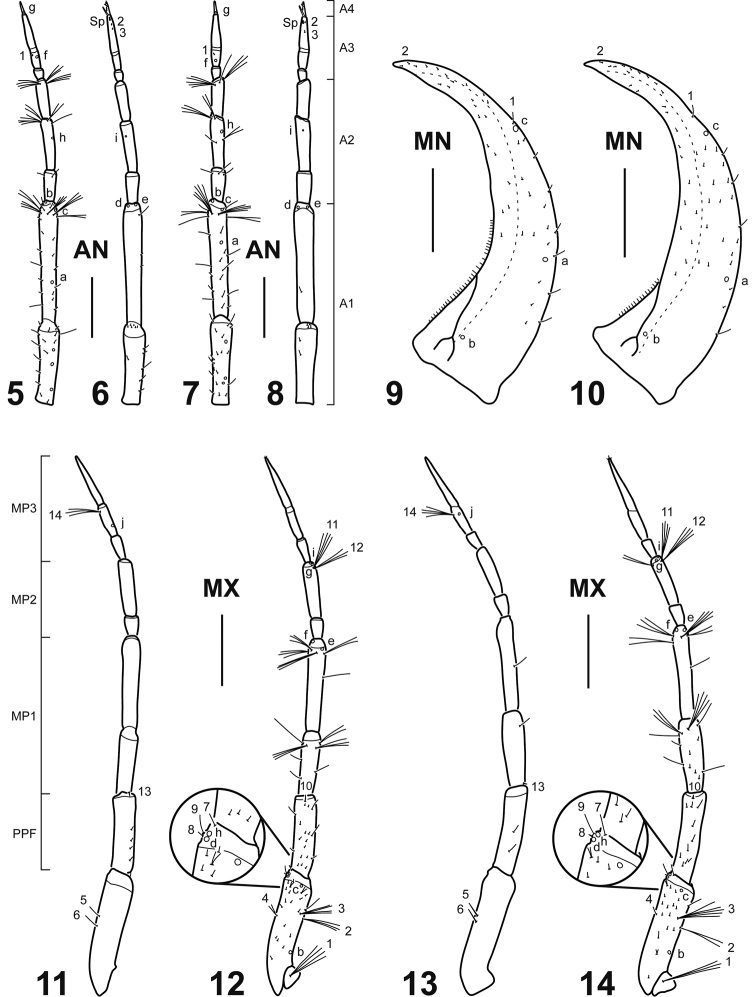
Head appendages of *Spencerhydrus* species, instar I **5***S. latecinctus*, right antenna, dorsal aspect **6***S. latecinctus*, left antenna, ventral aspect **7***S. pulchellus*, right antenna, dorsal aspect **8***S. pulchellus*, left antenna, ventral aspect **9***S. latecinctus*, right mandible, dorsal aspect **10***S. pulchellus*, right mandible, dorsal aspect **11***S. latecinctus*, right maxilla, dorsal aspect **12***S. latecinctus*, left maxilla, ventral aspect **13***S. pulchellus*, right maxilla, dorsal aspect **14***S. pulchellus*, left maxilla, ventral aspect. Numbers and lowercase letters indicate primary setae and pores, respectively. Additional setae and pores not labelled. Abbreviations: A1–4: antennomeres 1–4; AN: antenna; MN: mandible; MP1–3: maxillary palpomeres 1–3; MX: maxilla; PPF: palpifer; SP: spinula. Scale bar: 0.30 mm.

**Figures 15–18.**
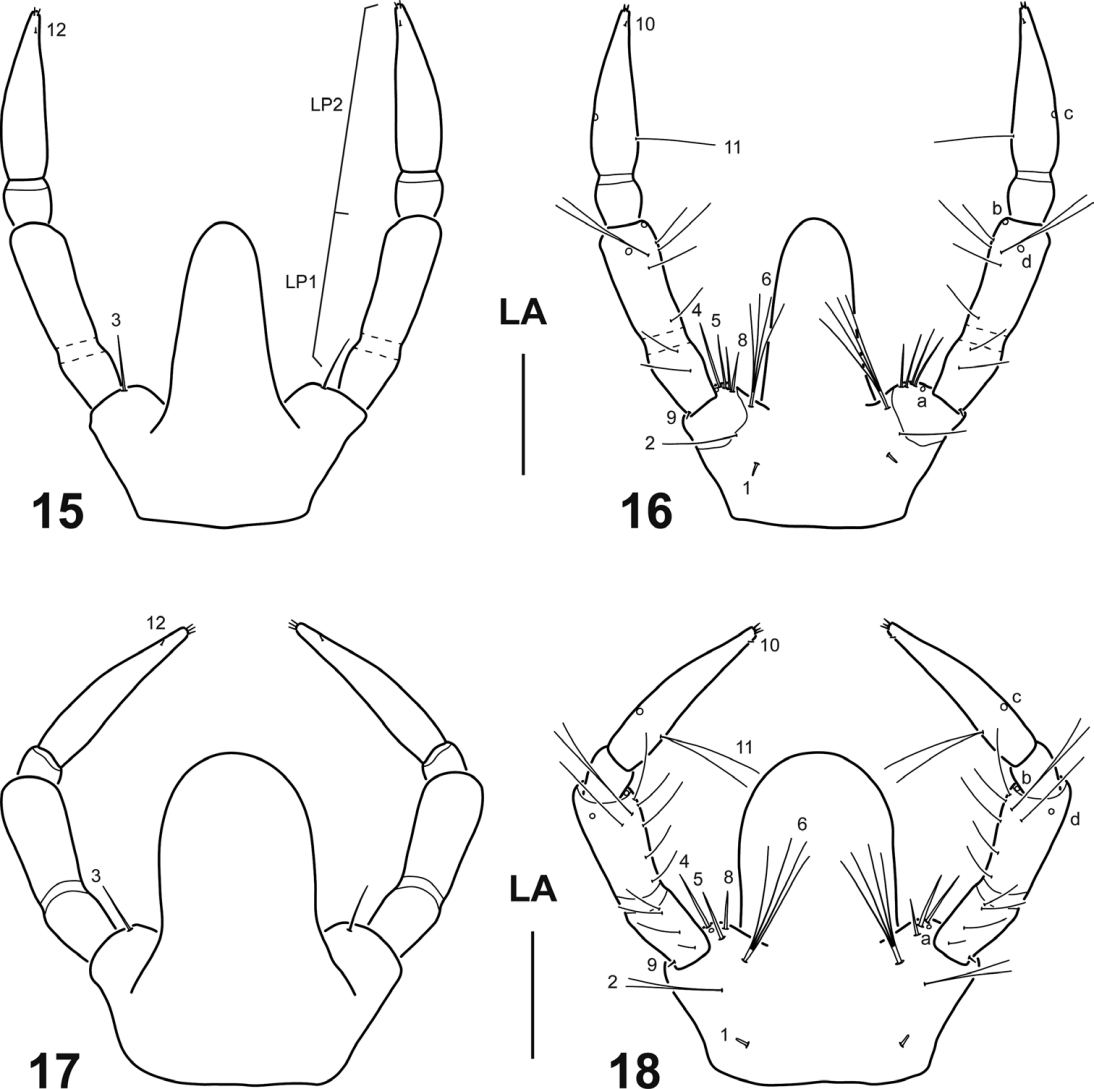
Labium of *Spencerhydrus* species, instar I **15***S. latecinctus*, dorsal aspect **16***S. latecinctus*, ventral aspect **17***S. pulchellus*, dorsal aspect **18***S. pulchellus*, ventral aspect. Numbers and lowercase letters indicate primary setae and pores, respectively. Additional setae and pores not labelled. Abbreviations: LA: labium; LP1–2: labial palpomeres 1–2. Scale bar: 0.15 mm.

**Figures 19–22.**
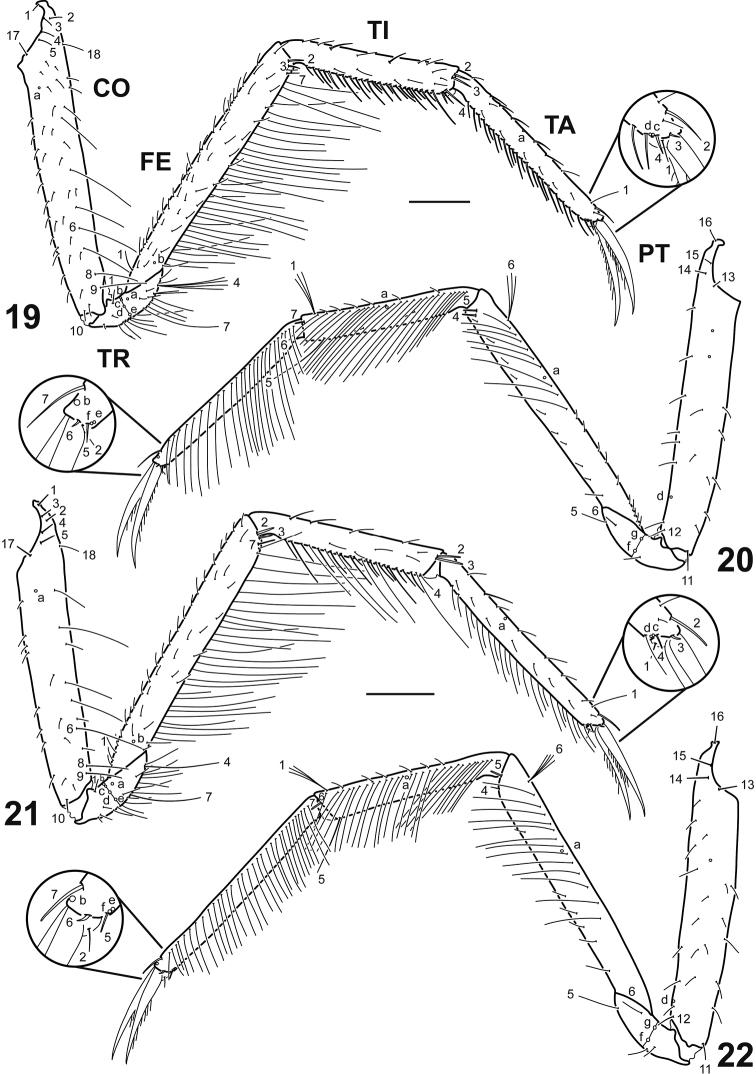
Left metathoracic leg of *Spencerhydrus* species, instar I **19***S. latecinctus*, anterior aspect **20***S. latecinctus*, posterior aspect **21***S. pulchellus*, anterior aspect **22***S. pulchellus*, posterior aspect. Numbers and lowercase letters indicate primary setae and pores, respectively. Additional setae and pores not labelled. Abbreviations: CO: coxa; FE: femur; PT: pretarsus; TA: tarsus; TI: tibia; TR: trochanter. Scale bar: 0.20 mm.

Larvae of *Spencerhydrus* can be distinguished from those of other Cybistrini genera by the following combination of characters: medial projection of frontoclypeus slightly indented apically, with lamellae clypeales directed forward in a characteristic V-shaped pattern (Figs 1, 2, 31, 32); lateral projections of frontoclypeus entire, with lamellae clypeales directed forward (Figs 1, 2, 31, 32); notches separating medial and lateral projections of frontoclypeus wide (Figs 1, 2, 31, 32); antennomere 1 with two or three additional pores proximally (Figs 5–7); mandible lacking crown of multifid setae on distal fourth (Figs 9, 10); maxillary palpomere 1 subdivided into two articles (Figs 11–14); median process of prementum strongly developed (Figs 15–18); prothorax with a single ventral sclerite; seta TI7 short, spine-like (Figs 20, 22); ventral row of setae on tibia and tarsus formed by setae of different lengths (Figs 19, 21); claws with basoventral spinulae (Figs 19–22); urogomphi very small but still longer than broad, included together with anus in the membranous ventrodistal area of abdominal segment VIII (Figs 25, 26); urogomphus bearing a single additional pore (Figs 27, 29).

**Instar I** (Figs 1–30). ***Color*.** Cephalic capsule pale yellow with small, irregular, light brown maculae on central portion of FR, on posterior half of PA dorsally, and few on central portion of PA ventrally (maculae weakly marked in some specimens); a light brown ring-like band present on neck region contiguous to occipital suture; AN, MX, and LA either completely pale yellow or with A4 and apices of A3 and MP3 light brown; MN with distal region light brown; thoracic tergites pale yellow, protergite with some small light brown maculae, meso- and metatergite each with four small light brown maculae marginally; abdominal tergites I–VI pale yellow, each with 4–6 small light brown maculae marginally; tergite VII pale yellow; sclerite of segment VIII uniformly pale yellow to light brown; membranous parts creamy white; legs pale yellow, distal portion and claws sometimes light brown; urogomphus light brown.

***Body*.** Elongate, subcylindrical. Measurements and ratios that characterize the body shape are shown in Table 1.

**Table 1.** Measurements and ratios for the larvae of *Spencerhydrus*.

**Measure**	***S. latecinctus***		***S. pulchellus***		
	**Instar I (*N* = 4)**	**Instar II (*N* = 1)**	**Instar I (*N* = 3)**	**Instar II (*N* = 4)**	**Instar III (*N* = 4)**
HL (mm)	2.55–2.63	3.73	2.14–2.28	2.93–3.14	3.94–4.40
HW (mm)	1.86–1.98	2.93	1.75–1.89	2.38–2.63	3.20–3.51
FRL (mm)	1.08–1.10	1.44	0.94–0.98	1.11–1.19	1.51–1.58
OCW (mm)	0.71–0.83	1.19	0.60–0.64	0.86–0.99	1.33–1.55
HL/HW	1.32–1.38	1.27	1.21–1.22	1.19–1.26	1.20–1.27
HW/OCW	2.33–2.70	2.46	2.92–2.96	2.47–2.84	2.27–2.42
COL/HL	0.57–0.59	0.61	0.56–0.57	0.62–0.63	0.62–0.65
FRL/HL	0.41–0.43	0.39	0.43–0.44	0.37–0.38	0.35–0.38
A/HW	1.07–1.13	0.97	1.08–1.16	0.93–1.06	0.84–0.92
A2/A1	0.63–0.69	0.58	0.58–0.60	0.56–0.60	0.52–0.59
A3/A1	0.34–0.37	0.28	0.32–0.34	0.31–0.32	0.28–0.31
A4/A3	0.18–0.22	0.15	0.23–0.24	0.15–0.17	0.13–0.14
A3’/A4	0.75–1.00	0.77	0.76–0.88	0.67–0.86	0.69–0.85
MNL/MNW	3.40–3.59	3.73	3.28–3.41	3.23–3.50	3.31–3.95
MNL/HL	0.48–0.50	0.51	0.54–0.55	0.51–0.54	0.50–0.52
A/MP	1.44–1.47	1.56	1.40–1.48	1.52–1.56	1.51–1.61
PPF/MP1	0.49–0.54	0.58	0.56–0.60	0.53–0.60	0.57–0.63
MP1/MP2	2.09–2.17	2.22	2.17–2.19	2.08–2.16	2.13–2.30
MP3/MP2	1.50–1.60	1.27	1.48–1.53	1.27–1.32	1.14–1.18
MP/LP	2.62–2.92	2.63	2.71–2.81	2.72–2.89	2.51–2.71
LP2/LP1	1.00–1.04	0.75	1.00–1.04	0.81–0.97	0.74–0.80
L3 (mm)	5.50–6.06	7.84	5.01–5.28	6.46–6.72	8.14–8.46
L3/L1	1.21–1.24	1.21	1.20–1.23	1.20–1.23	1.20–1.23
L3/L2	1.10–1.13	1.12	1.09–1.12	1.10–1.12	1.10–1.12
L3/HW	2.95–3.07	2.68	2.80–2.87	2.56–2.73	2.40–2.54
L3 (CO/FE)	1.04–1.15	1.11	1.00–1.05	1.04–1.07	1.04–1.10
L3 (TI/FE)	0.64–0.68	0.65	0.65–0.67	0.62–0.63	0.60–0.62
L3 (TA/FE)	0.75–0.78	0.65	0.77–0.80	0.67–0.72	0.60–0.64
L3 (CL/TA)	0.44–0.53	0.45	0.44–0.47	0.40–0.48	0.37–0.45
LAS (mm)	3.45–3.58	5.09	3.00–3.23	3.99–4.04	5.38–5.70
LAS/HW	1.79–1.88	1.74	1.69–1.73	1.54–1.68	1.54–1.68
U (mm)	0.09–0.10	0.12	0.11–0.12	0.08–0.10	0.10–0.11

***Head*.** Cephalic capsule (Figs 1–4). Flattened, subtriangular, longer than broad; maximum width at level of stemmata, constricted at level of occipital region; occipital suture present; ecdysial suture well marked, coronal line long; occipital foramen deeply emarginate ventrally; posterior tentorial pits close to well visible ventral midline; FR subtriangular, anterior margin divided into three well developed projections: medial projection broad, well projected forward, slightly indented apically; lateral projections broad, rounded, less projected forward; anterolateral lobes rounded, not projected beyond anterior margin; egg bursters large, rounded to somewhat pointed, located laterally close to ecdysial line; four stemmata on upper side of head and two on underside, arranged in two vertical series. *Antenna* (Figs 5–8). Elongate, slender, somewhat longer than HW, composed of four antennomeres; A1 longest, subdivided into two articles, distal one somewhat less than twice longer than basal one; A2 shorter than A1, subdivided into three articles, basal one shortest, medial one longest; A3 shorter than A2, subdivided into three articles, basal one shortest, distal one longest, bearing a ventroapical spinula; apical lateroventral process of A3 slender, elongate; A4 shortest, with a small lateroventral process on distal third. *Mandible* (Figs 9, 10). Strong, falciform, broad at base, narrowing to acute apex, more abruptly narrowed on distal fourth; mandibular channel present. *Maxilla* (Figs 11–14). Premaxillary lobes well developed; cardo sub-ovate to subtriangular; stipes elongate, slender, subcylindrical; galea absent; PPF elongate, palpomere-like; MP elongate, slender, shorter than antenna, composed of three palpomeres, MP1 longest, MP2 shortest; MP1 and MP2 subdivided into two articles, distal one longer than basal one; MP3 subdivided into three articles, basal one shortest, distal one longest. *Labium* (Figs 15–18). Prementum broader than long, anterodorsal margin projected forward into strongly developed, apically rounded median process; LP much shorter than MP, composed of two palpomeres subequal in length; LP1 more or less weakly subdivided into two articles, distal one somewhat longer than basal one; LP2 subdivided into two articles, distal one much longer than basal one.

***Thorax*.** Terga convex, pronotum shorter than subequal meso- and metanotum combined; protergite subrectangular, margins somewhat truncate, much more developed than meso- and metatergite; meso- and metatergite small, subrectangular to subtrapezoidal with posterior margin indented; all three tergites with sagittal line, lacking anterotransverse carina; sterna membranous except for a single sclerite on anterior portion of prothorax; spiracles absent. *Legs* (Figs 19–22). Long, composed of six articles, L1 shortest, L3 longest; CO elongate, subcylindrical, TR divided into two parts by an annulus, FE, TI and TA slender, subcylindrical, PT with two long, slender, slightly curved claws, anterior claw slightly longer than posterior claw on L1 and L2, claws subequal in length on L3; proTA with a row of well-developed, bifid, ventral spinulae, those on basal third shorter, multifid, forming a dense patch (cleaning device); claws with elongate ventral spinulae on basal 1/2 to basal 2/3.

**Figures 23–26.**
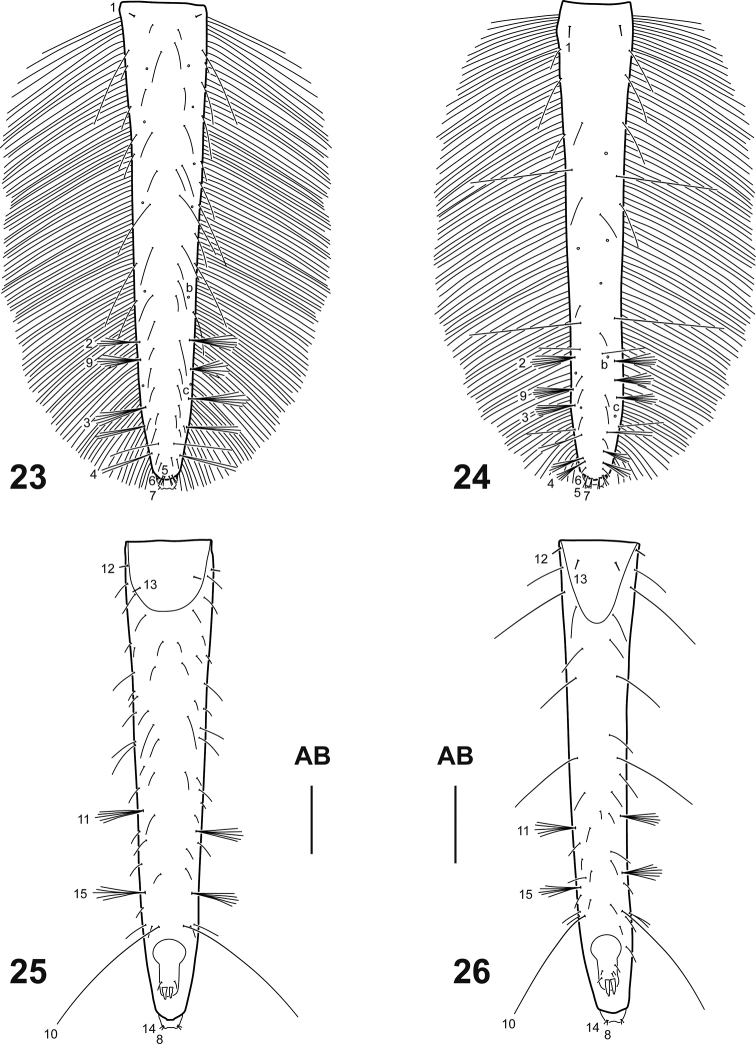
Abdominal segment VIII of *Spencerhydrus* species, instar I **23***S. latecinctus*, dorsal aspect **24***S. pulchellus*, dorsal aspect **25***S. latecinctus*, ventral aspect **26***S. pulchellus*, ventral aspect. Numbers and lowercase letters indicate primary setae and pores, respectively. Additional setae and pores not labelled. Abbreviation: AB: abdominal segment VIII. Scale bar: 0.50 mm.

**Figures 27–30.**
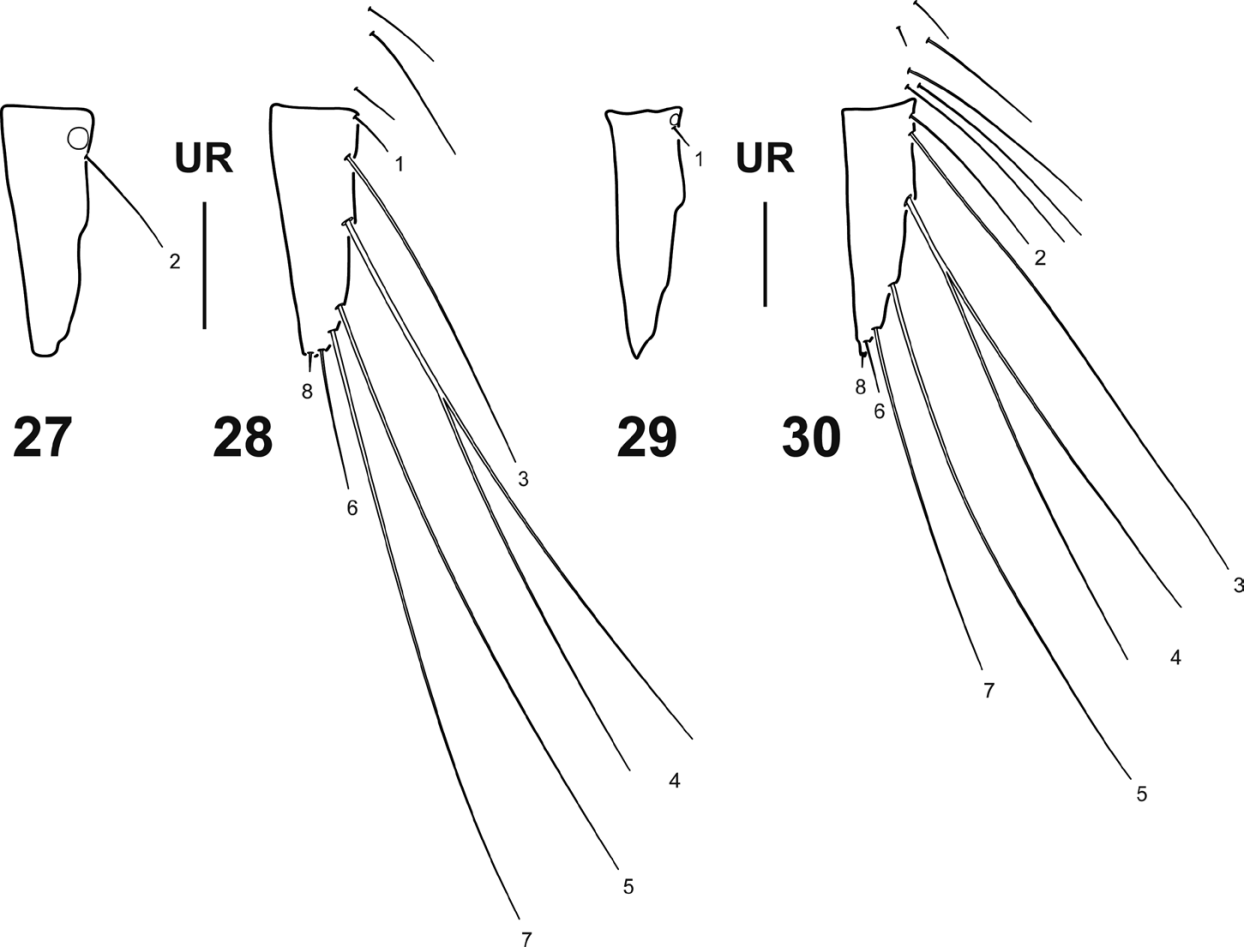
Urogomphus of *Spencerhydrus* species, instar I **27***S. latecinctus*, right urogomphus, dorsal aspect **28***S. latecinctus*, left urogomphus, ventral aspect **29***S. pulchellus*, right urogomphus, dorsal aspect **30***S. pulchellus*, left urogomphus, ventral aspect. Numbers and lowercase letters indicate primary setae and pores, respectively. UR: urogomphus. Scale bar: 0.05 mm.

***Abdomen*.** Eight-segmented; segments I–VI subequal, membranous except for a small anterodorsal sclerite and a minute sclerite on each lateral; tergites I–VI subrectangular, lacking anterotransverse carina, sagittal line visible, posterior half covered with short spinulae; segment VII narrower, main sclerite covering about anterior half of dorsal surface, lacking anterotransverse carina, covered with short spinulae, sagittal line not found; segments I–VII with a minute lateral sclerite, lacking spiracles; segment VIII (= LAS, Figs 23–26) longest and narrowest, completely sclerotized except ventrodistally around anus and urogomphi, lacking anterotransverse carina and sagittal line, basal half covered with short spinulae; siphon short. *Urogomphus* (Figs 27–30). Strongly reduced, approximately two or three times longer than broad, composed of one urogomphomere.

***Chaetotaxy*** (Figs 1–30). Similar to that of generalized Cybistrini larva (Alarie et al. 2011) with the following remarks: FR with numerous lamellae clypeales distributed on the apices of the anterior projections, those on medial projection longer than the others, arranged in a characteristic V-shaped pattern; A1 with some additional multifid setae distally and two or three additional pores proximally; crown of elongate additional (usually multifid) setae on distal fourth of MN absent; seta LA8 present; ventral row of setae on TI and TA formed by setae of different lengths; setae CO7, TR2, TR3, FE8, FE9, FE10, and pore ABa most likely present but obscured by the presence of additional sensilla; seta AB4 bifid or multifid; U bearing all ancestral setae and one pore; several short additional setae present on membranous area of abdominal segment VIII, near urogomphal base.

**Figures 31–32.**
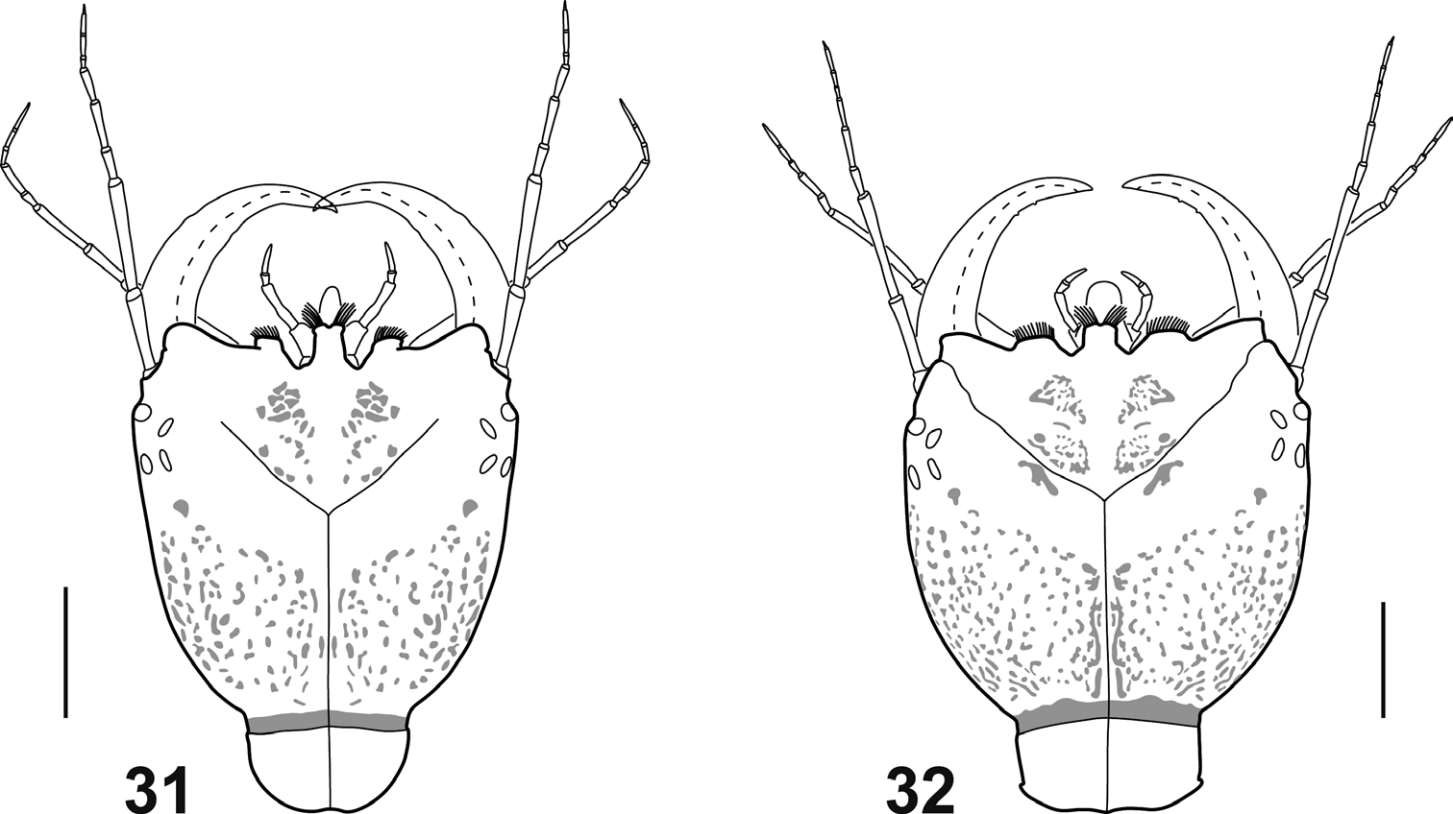
Head of *Spencerhydrus* species **31***S. latecinctus*, instar II, dorsal aspect **32***S. pulchellus*, instar III, dorsal aspect. Scale bar: 1.00 mm.

**Instar II** (Fig. 31). As instar I except for the following features:

***Color*.** Light brown maculae on cephalic capsule more numerous; thoracic tergites with some light brown maculae on disc; maculae on abdominal tergites I–VI more extended; sclerite of segment VII with some small light brown maculae.

***Body*.** Measurements and ratios that characterize the body shape are shown in Table 1.

***Head*** (Fig. 31). Egg bursters absent; A subequal in length to HW; LP1 somewhat longer than LP2.

***Thorax*.** Anterotransverse carina present on metatergite, present or absent on mesotergite; spinulae of claws restricted to middle region.

***Abdomen*.** Tergites I–VI with anterotransverse carina, sometimes weakly marked; sclerites I–VIII lacking spinulae.

***Chaetotaxy*.** Secondary setae most likely present on different body parts, although difficult to evaluate due to large number of additional setae.

**Instar III** (Fig. 32). As instar II except for the following features:

***Color*.** Somewhat darker in general; abdominal tergites I–VII predominantly light brown with longitudinal creamy white area centrally.

***Body*.** Measurements and ratios that characterize the body shape are shown in Table 1.

***Head*** (Fig. 32). Somewhat more robust and parallel sided. Antenna shorter than HW.

***Thorax*.** Meso- and metatergite covering most of dorsal surface; well-developed spiracles present on mesothorax.

***Abdomen*.** Well-developed spiracles present on segments I–VII.

### *Spencerhydrus latecinctus* Sharp, 1882

**Source of material.** The descriptions of this species are based on nine specimens of instar I and one of instar II (no instar III was available). Larvae were collected in Australia at the following localities: 1) SW Victoria, Kinhil, 14.x.1983 and 9.xi.1983; 2) SA, Watervalley SE 8 km N Mt. Rough, 15.x.2000; 3) SA, 6 km W Penola, 30.x.2001. The association is firm as *S. latecinctus* is the only species of *Spencerhydrus* in south-east Australia, and the larvae of the other Cybistrini genera potentially present in the area (*Cybister* Curtis, 1827, *Onychohydrus* and *Sternhydrus*) clearly differ morphologically from the studied material (Alarie et al. 2011; Michat et al. 2015).

**Differs from *S. pulchellus* as follows.** Larger size (Table 1); median process of prementum relatively slender (Figs 15, 16); anterior margin of prementum with one additional seta (Fig. 16); setae LA2 and LA11 unified (Fig. 16); additional setae on ventral margin of meso- and metatibia more robust, dissimilar in length but not much so (Fig. 19); ratio LAS/HW (Table 1).

### *Spencerhydrus pulchellus* Sharp, 1882

**Source of material.** The descriptions of this species are based on nine specimens of instar I, four of instar II, and four of instar III. Larvae were collected in Australia at the following localities: 1) WA, Ellenbrook Nat. Res., 14.ix.2000; 2) WA, 6 km S Pinjarra, 23.x.1996, 23.ix.2000, and 3.x.2003. The association is firm as *S. pulchellus* is the only species of *Spencerhydrus* in south-west Australia, and the larvae of the other Cybistrini genera potentially present in the area (*Cybister* and *Onychohydrus*) clearly differ morphologically from the studied material (Alarie et al. 2011).

**Differs from *S. latecinctus* as follows.** Smaller size (Table 1); median process of prementum relatively broader (Figs 17, 18); anterior margin of prementum lacking additional setae (Fig. 18); setae LA2 and LA11 bifid (Fig. 18); additional setae on ventral margin of meso- and metatibia less robust and highly dissimilar in length (Fig. 21); ratio LAS/HW (Table 1).

## Discussion

Similarly to all other members of the tribe Cybistrini known with sufficient chaetotaxic detail (Michat 2006, 2010; Alarie et al. 2011; Michat et al. 2015), larvae of *Spencerhydrus* are characterized by bearing a large number of additional setae on almost all body regions. This particularly high number of additional setae (with many of them multifid or variously modified) distinguishes members of this tribe from all other known diving beetles, and summed to the very large size of cybistrine larvae somewhat justifies the title’s opening statement. An exception to this pattern, however, is seen in the urogomphi. The miniaturization of this structure within Cybistrini, although showing a different degree of reduction among genera (see Alarie et al. 2011), may have prevented the development of additional sensilla, to the point that they bear the same number of (or even less) setae than in other dytiscids, and a highly reduced number of pores.

As mentioned above (see Introduction) the genus *Spencerhydrus* was included in recent phylogenetic analyses of the Cybistrini (Michat et al. 2015) and Dytiscidae (Michat et al. 2017) based on larval morphology. Most of the characters supporting monophyly and relationships of this genus are corroborated in our study, and therefore we find worth mentioning them. *Spencerhydrus* was resolved as part of a clade of Australian Cybistrini together with *Onychohydrus* and *Sternhydrus*, and within this clade, it is sister to the clade formed by the other two genera. Synapomorphies in support of a clade *Spencerhydrus* + *Onychohydrus* + *Sternhydrus* are the presence of additional pores on antennomere 1, the subdivision of the maxillary palpomere 1 into two articles, the short and spine-like aspect of the tibial seta TI7, the presence of very small urogomphi, although still longer than broad, included together with anus in the non-sclerotized ventrodistal area of abdominal segment VIII, and the absence of a crown of multifid setae on the distal fourth of mandible (this last character shared with members of the subgenus Trifurcitus Brinck, 1945 of *Megadytes* Sharp, 1882). On the other hand, the following autapomorphies characterize the genus *Spencerhydrus* within Cybistrini: medial projection of frontoclypeus slightly indented apically, with lamellae clypeales directed forward in a characteristic V-shaped pattern (Figs 1, 2); median process of prementum strongly developed (Figs 15–18); presence of a single ventral sclerite on prothorax; and presence of basoventral spinulae on claws (Figs 19–22). Watts (2002), in his key to larvae of the Australian genera of Dytiscidae, gives the reduced sclerotization (covering only anterior half) of the abdominal segment VII as diagnostic for *Spencerhydrus*. This is another good character to separate the genus from the other Cybistrini because, given the large size of the larvae, it is easily visible under low magnifications.

Although very similar morphologically, we were able to find some characters to confidently separate larvae of the two species of *Spencerhydrus* (see earlier). The most conspicuous of these characters is the shape of the median process of prementum, which is visibly broader in *S. pulchellus* than in *S. latecinctus* (compare Figs 15 and 16 with Figs 17 and 18). This difference seems to be constant between larval instars, as the comparison of the second instar of both species offers the same picture. Unfortunately, the third instar of *S. latecinctus* was not available for comparison, but we estimate that this difference most likely remains the same in this stage.

## Supplementary Material

XML Treatment for Spencerhydrus
